# Meta-Analysis of the Association between Insulin-Like Growth Factor Binding Protein 3 Genetic Polymorphisms and Colorectal Cancer Susceptibility

**DOI:** 10.1371/journal.pone.0059665

**Published:** 2013-03-20

**Authors:** Hao Xiang, Ying Wang, Shaofa Nie

**Affiliations:** 1 Department of Epidemiology and Biostatistics, School of Public Health, Wuhan University, Wuhan, China; 2 Global Health Institute, Wuhan University, Wuhan, China; 3 Department of Epidemiology and Biostatistics, and the Ministry of Education Key Lab of Environment and Health, School of Public Health, Tongji Medical College, Huazhong University of Science and Technology, Wuhan, China; Cardiff University, United Kingdom

## Abstract

Insulin-like growth factor binding protein 3 (IGFBP-3) plays an important role in the development and progress of cancers. The association between *IGFBP-3* polymorphisms and colorectal cancer remains controversial and ambiguous. The aim of this study is to explore the association between *IGFBP3* A-202C and Gly32Ala polymorphisms and colorectal cancer susceptibility using meta-analyisi. Case-control studies on the association between *IGFBP3* A-202C and Gly32Ala polymorphisms and colorectal cancer, which had sufficient data for estimating an odds ratio (OR) with 95% confidence interval (CI), were included in the meta-analysis. Abstracts, case reports, editorials, and review articles were excluded. Heterozygous and homozygous mutants were compared with the wild types to estimate combined *OR* values and 95%*CIs* with Review Manager 5.0. Six eligible studies were included, with 3157 patients and 6027 controls for A-202C and 1711 patients and 2995 controls for Gly32Ala. No significant association was found in all genetic models (for A-202C, AC vs. AA, OR = 0.99(0.88–1.11), CC vs. AA, OR = 1.06(0.92–1.22), dominant model, OR = 0.98(0.88–1.09), recessive model, OR = 0.94(0.84–1.05); and for Gly32Ala polymorphism, GC vs. GG, OR = 1.10(0.92–1.31), CC vs. GG, OR = 0.93(0.76–1.14), dominant model, OR = 1.05(0.89–1.24), recessive model, OR = 0.90(0.77–1.05)). The results suggest that the *IGFBP3* A-202C and Gly32Ala polymorphisms are not associated with colorectal cancer susceptibility.

## Introduction

Colorectal cancer is the third most common diagnosed cancer in males and the second in females, with over 1.2 million new cancer cases and 608,700 deaths worldwide in 2008 [1], [2]. Genetic susceptibility to this disease may result from inherited mutations in genes involved in proliferation and apoptosis.

The insulin-like growth factor (IGF) family, including insulin-like growth factor 1 (IGF1), insulin-like growth factor 2 (IGF2)and insulin-like growth factor binding protein (IGFBP), are involved in proliferation and apoptosis, and thus play a significant role in both normal and malignant cell growth [3]. In the circulation, about 90% of IGF1 is bound to IGFBP3, which regulates the distribution and bioavailability of IGF1 [4]. In addition, IGFBP3 exerts anti-proliferative and apoptotic effects that are mediated through a specific cell surface receptor [5]. Epidemiological studies show that high levels of IGF1 and low levels of IGFBP3 are associated with an increased risk for several common cancers, including cancer of the prostate, breast, lung, and colorectum [6]–[8]. Although many personal and lifestyle factors, including body mass index (BMI), vigorous physical activity and smoking, may affect the circulating levels of IGFBP3 [9], a twin study demonstrated that heritable factors may account for 60% of the inter-individual variation in IGFBP3 levels [10].

Two genetic polymorphisms have been identified as influencing the circulating levels of IGFBP3. One is a promoter single nucleotide polymorphism (SNP) located at position -202 (rs2854744, A>C) a transcription start site that is believed to affect the promoter activity [11]. The other polymorphism is a non-synonymous substitution, Gly32Ala (rs2854746, G>C), a site for high affinity binding of IGF1 [12]. The presence of the variant 32Ala allele was inversely associated with IGFBP3 levels [13].

Until 2009, there were several studies evaluating associations between the IGFBP3 polymorphisms and cancer risk in diverse populations and in multiple types of cancer, but their outcomes have been contradictory. Li et al’s meta-analysis showed significant association was found in additive genetic model between IGFBP3 A-202C SNP and breast cancer and prostate cancer [14], Chen et al’s meta-analysis suggest IGFBP3 202CC genotype was associated with an increased risk of prostate cancer with borderline significance [15]. However, it is hard to explore the association between IGFBP3 SNPs and colorectal cancer because there are only 3 papers to investigate this issue before 2009, another two articles(Xiang et al [16] and Feik at al [17]) exploring this issue were published in 2009 and 2010, however, these results are contrary than conclusive, so we think it is meaningful to estimate the effect of genotypes of *IGFBP3* on risk for colorectal cancer.

## Materials and Methods

### Identification and Eligibility of Studies

To identify all articles that explored the association of *IGFBP3* A-202C and Gly32Ala polymorphisms with colorectal cancer, we conducted a literature search of the PubMed database (last search on December 31st, 2011) using the following search terms: ‘IGFBP3’ or ‘Insulin growth factor binding protein 3′, ‘polymorphism’, and ‘colorectal cancer’. The search followed the guidelines of the 2009 preferred reporting items for systematic reviews and meta-analysis (PRISMA) statement (Table S1). All eligible articles were retrieved and their references were checked for other relevant articles. Abstracts, case reports, editorials, and review articles were excluded. All the studies included had to meet all the following criteria: (1) case-control design; (2) outcome of colorectal cancer; (3) sufficient data for estimating an odds ratio (OR) with 95% confidence interval (CI).

### Data Extraction

Data were carefully extracted from all eligible publications independently by two investigators (Hao X and Ying W). For conflicting evaluation, an agreement was reached following discussion. For each study, the following characteristics were collected: first author, year of publication, control groups studied, ethnicity, genotypes and allele frequency of cases and controls.

### Statistical Analysis

The meta-analysis examined the overall association of allele C of A-202C site and the risk for colorectal cancer, including the comparisons of co-dominant (CC vs. AA, CA vs. AA), recessive (CC vs. AC+AA) and dominant (CC+AC vs. AA) models. The same comparisons were performed for Gly32Ala allele C to G. The summary odds ratios (OR) and 95% confidence intervals (CIs) were used to assess the strength of association. DerSimonian and Laird Q test was used to assess the degree of heterogeneity between studies and the heterogeneity was considered significant when *P*<0.05 [18], [19]. Fixed-effect model, based on the Mantel–Haenszel method, was used when no significant heterogeneity among the studies was found (*P*>0.05). Otherwise, a random-effect model was chosen.

Publication bias was investigated by funnel plot, in which the standard error of log (OR) of each study was plotted against its OR. Funnel plot asymmetry was assessed by Egger’s test, a linear regression approach to measure asymmetry on the natural logarithm scale of the OR [20]. The departure from the Hardy-Weinberg equilibrium for the control group in each study was assessed with Pearson's goodness-of-fit chi-square test with 1 degree of freedom.

All data were analyzed using Statistical Analysis System software (v.9.1.3; SAS Institute, Cary, NC, USA), STATA7.0 (Stata-Corp, College Station, TX, USA) and Review Manager (v.5.0, Oxford, England). All *p*-values were based on two-sided tests and a *p*-value of less than 0.05 was considered statistically significant.

## Results

**Figure 1:**
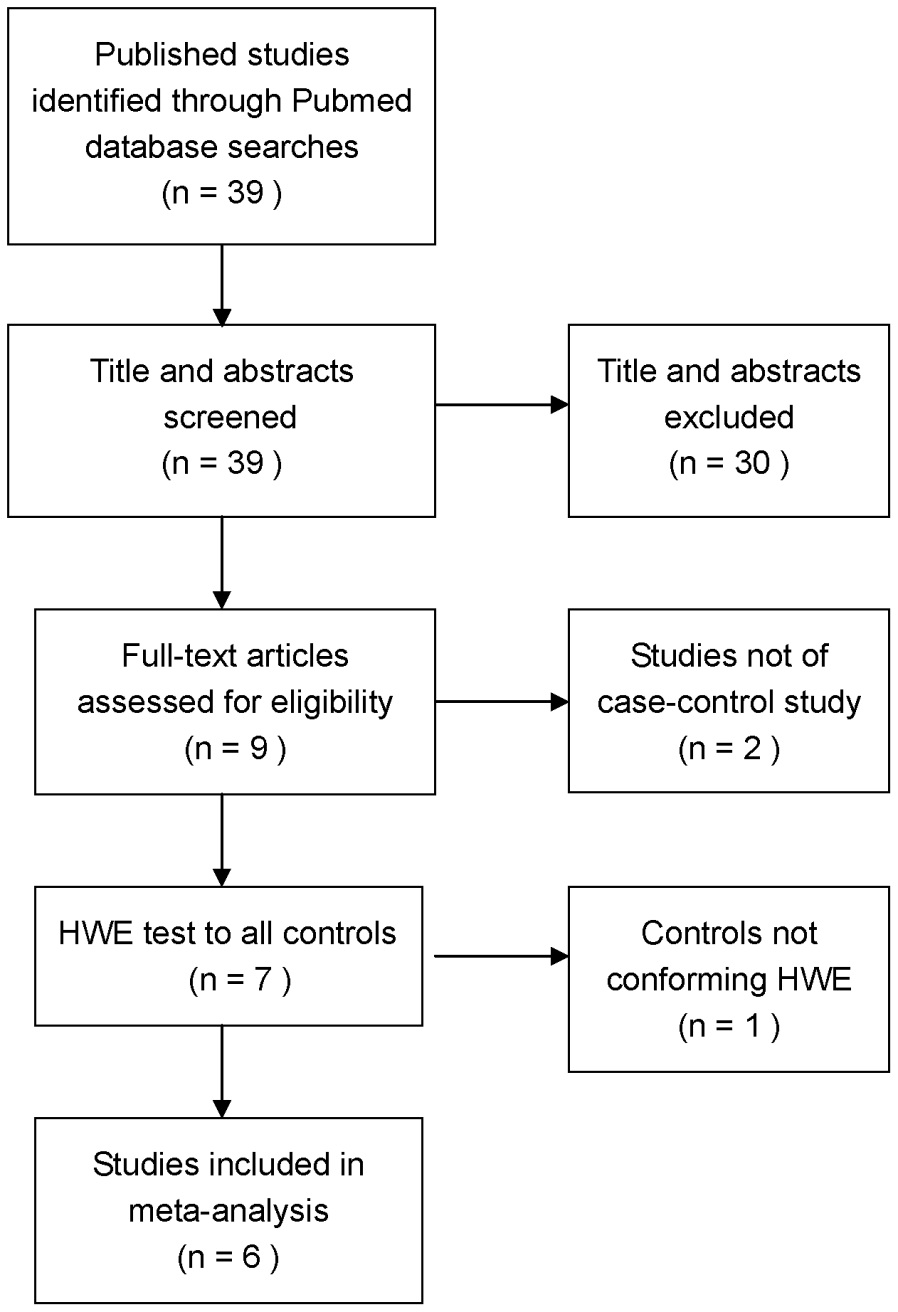
The process of identifying relevant studies is summarized.

From 39 publications identified by initial data searches, nine studies examining the association of *IGFBP3* -A202C and Gly32Ala polymorphisms with colorectal cancer were identified (Figure 1). Two articles were excluded because they were not case control studies. In one study [21], the distribution of genotypes in controls was not consistent with Hardy-Weinberg equilibrium, so it was also excluded. Six published studies were eligible for further analysis, including 4 population-based and 2 hospital-based case control studies. As shown in Table 1, 5 of the studies [13], [16], [22]–[24] evaluated the relationship between *IGFBP3* -A202C polymorphisms and colorectal cancer and included 3157 cases and 6027 controls. Meanwhile, 4 studies [13], [16], [17], [23] evaluated the relationship between *IGFBP3* Gly32Ala polymorphisms and colorectal cancer and included 1711 cases and 2995 controls.

**Table 1:** Distribution of genotypes and alleles of IGFBP3 A-202C and Gly32Ala polymorphisms among cases and controls.

SNP	Author(year)	Type^a^	Ethnicity^b^	Case					Control				
				AA (%)	AC (%)	CC (%)	A (%)	C (%)	AA (%)	AC (%)	CC (%)	A (%)	C (%)
A-202C	Slattery(2004)	PB	C	427(21.9)	997(51.2)	524(26.9)	1851(47.6)	2045(52.5)	463(21.5)	1082(50.3)	607(28.2)	2008(46.7)	2296(53.3)
	Wong(2005)	PB	A	166(61.0)	90(33.1)	16(5.9)	422(77.6)	122(22.4)	480(58.5)	306(37.3)	35(4.3)	1266(77.1)	376(22.9)
	Pechlivanis(2007)	PB	C	135(22.0)	314(51.1)	165(26.9)	584(47.6)	644(52.4)	122(22.3)	262(47.9)	163(29.8)	506(46.3)	588(53.7)
	Xiang(2009)	HB	A	121(59.9)	69(34.2)	12(5.9)	311(77.0)	93(23.0)	134(63.2)	68(32.1)	10(4.7)	336(79.2)	88(20.8)
	Feik(2010)	HB	C	37(19.8)	59(35.5)	32(34.7)	133(42.6)	109(57.4)	504(29.1)	845(48.8)	381(22.0)	1853(53.6)	1607(46.4)
				**GG (%)**	**GC (%)**	**CC (%)**	**G (%)**	**C (%)**	**GG (%)**	**GC (%)**	**CC (%)**	**G (%)**	**C (%)**
Gly32Ala	Morimoto(2005)	PB	C	173(22.1)	361(46.2)	248(31.7)	707(45.2)	857(54.8)	95(18.9)	226(44.9)	182(36.2)	416(41.4)	590(58.6)
	Pechlivanis(2007)	PB	C	111(18.3)	317(52.3)	178(29.4)	539(44.5)	673(55.5)	114(20.7)	259(47.1)	177(32.2)	487(44.3)	613(55.7)
	Xiang(2009)	HB	A	101(50.0)	84(41.6)	17(8.4)	286(70.8)	118(29.2)	129(60.8)	72(34.0)	11(5.2)	330(77.8)	94(22.2)
	Feik(2010)	HB	C	24(19.8)	55(45.5)	42(34.7)	103(42.6)	139(57.4)	320(18.5)	822(47.5)	588(34.0)	1462(42.3)	1998(57.7)

a Type of control source: ‘PB’ for population based, ‘HB’ for hospital based.

b Ethnicity: ‘C’ for Caucasian ancestry, ‘A’ for Asian ancestry.

**Figure 2:**
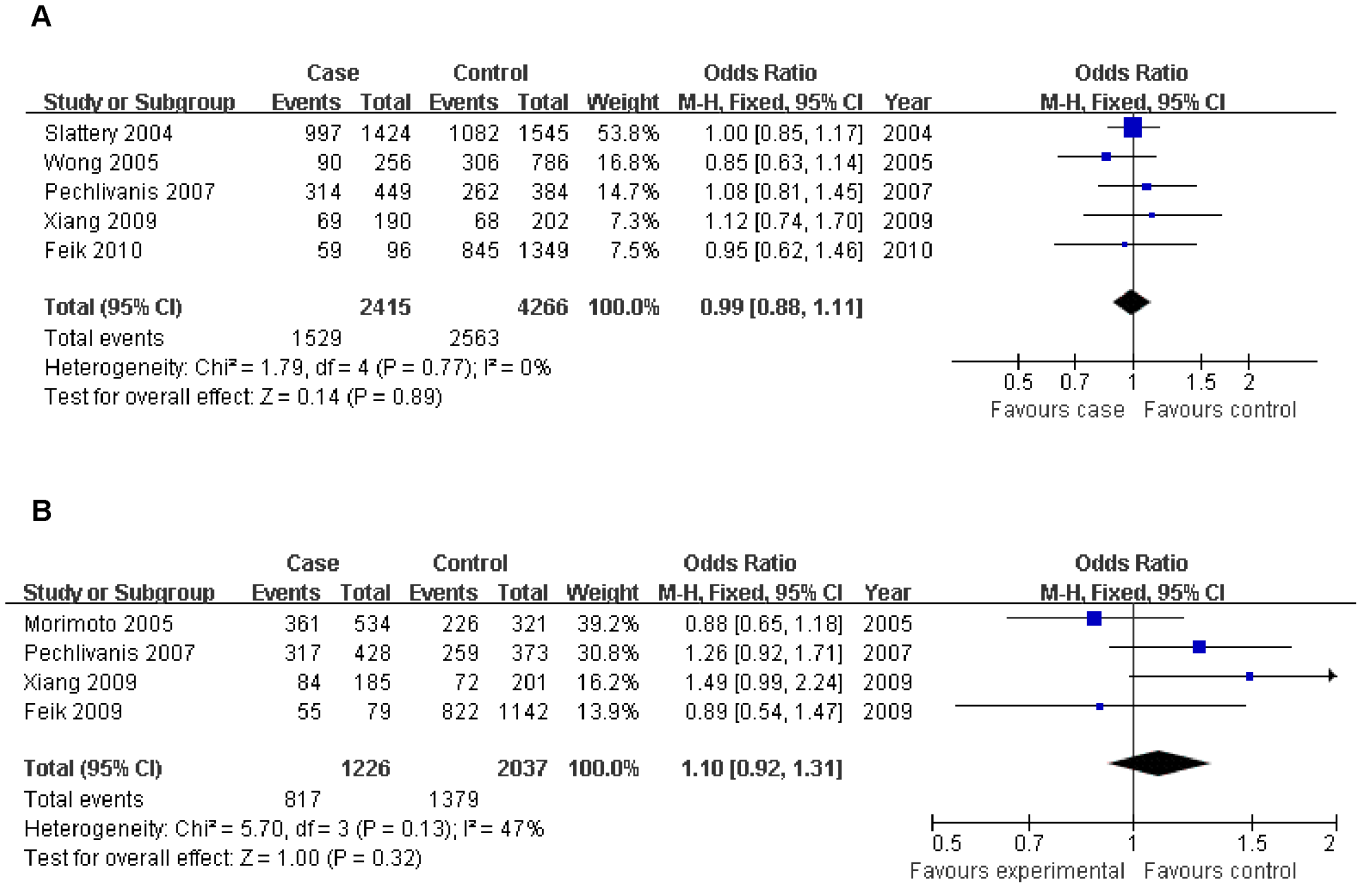
Association of IGFBP3 A-202C and Gly32Ala polymorphisms with colorectal cancer risk. Each comparison was presented by the year of publication. Part A analyzed the comparison between IGFBP3 A-202C(AC vs. AA) and colorectal cancer, par B analyzed the comparison between Gly32Ala polymorphism (GC vs. GG) and colorectal cancer.

For *IGFBP3* -A202C, there is no significant association with colorectal cancer risk when all studies are pooled into a meta-analysis (CA vs. AA: OR = 0.99, 95% CI = 0.88–1.11; CC vs. AA: OR = 1.06, 95% CI = 0.92–1.22; dominant model: OR = 0.98, 95% CI = 0.88–1.09; recessive model: OR = 0.94, 95% CI = 0.84–1.05) (Table 2). For the additive model, individuals carrying the C allele were not at increased risk for colorectal cancer (OR = 0.97, 95% CI = 0.91–1.04) (Figure 2A). There is no significantly elevated colorectal cancer risk in any genetic model when all studies are pooled into the analysis (CG vs. GG: OR = 1.10, 95% CI = 0.96–1.25; CC vs. GG: OR = 1.06, 95% CI = 0.82–1.37; dominant model: OR = 1.06, 95% CI = 0.88–1.27; recessive model: OR = 0.89, 95% CI = 0.80–1.01).

**Table 2:** Results of meta-analysis for *IGFBP3* A-202C/Gly32Ala polymorphism and colorectal cancer.

Genotype Comparisons	OR (95% C.I.)	*P*	*P_h_*	*I^2^*
A-202C				
AC vs. AA	0.99(0.88–1.11)	0.89	0.77	0%
CC vs. AA	1.06(0.92–1.22)	0.40	0.62	0%
CC+AC vs. AA	0.98(0.88–1.09)	0.71	0.89	0%
CC Vs. AC+AA	0.94(0.84–1.05)	0.28	0.63	0%
Gly32Ala				
GC vs. GG	1.10(0.92–1.31)	0.32	0.13	47%
CC vs. GG	0.93(0.76–1.14)	0.49	0.13	46%
CC+GC vs. GG	1.07(0.81–1.42)	0.62	0.05	61%
CC Vs. GC+GG	0.90(0.77–1.05)	0.17	0.31	16%

*P_h_ P* value of Q test for heterogeneity.

**Figure 3:**
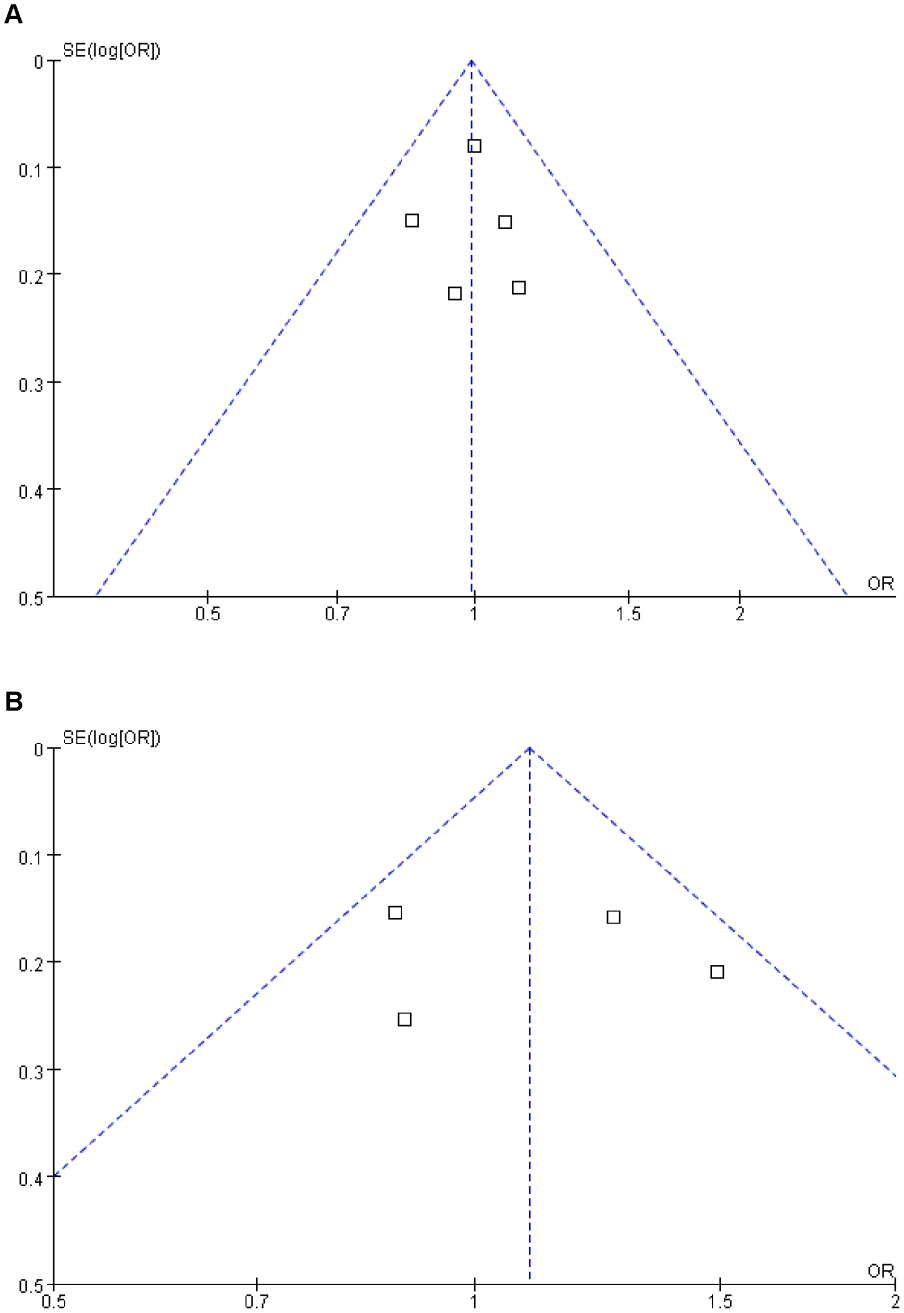
Funnel plots of IGFBP3 A-202C and Gly32Ala polymorphism and colorectal cancer risk. Part A, model: A-202C (AC Vs AA), *t*_eager’s test_ = 1.45, P_ eager’s test_ = 0.28. Part B, model: Gly32Ala (GC Vs GG), *t*_eager’s test_ = 0.76, P_ eager’s test_ = 0.35.

Sensitivity analysis was performed by sequential omission of individual studies for each comparison in multiple models. The results did not change the overall effects of the two SNPs on cancer risk under different genetic models, indicating that the significance of pooled ORs was not excessively influenced by any single study. The Funnel plot’s shapes of all comparisons did not reveal obvious evidence of asymmetry, and the results of Egger’s test also suggested that there was no evidence of publication bias. For example, as shown in Figure 3, the shape of the funnel plots does not reveal any evidence of obvious asymmetry, and results of Egger’s test did not suggest any evidence of publication bias (t = 1.45, *P* = 0.28 for A-202C, t = 0.76, *P* = 0.35 for Gly32Ala).

## Discussion

In this study, we employed a meta-analysis to provide the assessment of the association between *IGFBP3* A-202C and Gly32Ala polymorphisms and colorectal cancer risk by critically reviewing 5 studies of the A-202C polymorphism (a total of 3157 cases and 6027 controls) and 4 studies of the Gly32Ala polymorphism (a total of 1711 cases and 2995 controls). Heterogeneity analysis and sensitivity analysis were also critically performed to ensure the reliability of this meta-analysis. This meta-analysis indicated that these two polymorphisms in *IGFBP3* are not significantly associated with risk for colorectal cancer.

Because IGFBP3 has an important role in tumor development, polymorphisms located in *IGFBP3* might be potential markers in the evaluation of exposure of target organs to endogenous IGFBP3 on cancer risk. One of the potential mechanisms may be that the variant (G to C substitution) of Gly32Ala causes an amino acid change from alanine to glycine of codon 32, reducing percent binding of IGFBP3 and leading to lower concentration of IGFBP3 in the circulation. Of the 4 articles included in this meta-analysis, only one study shows that participants carrying the Gly32Ala GC heterozygote or CC homozygote have a significantly increased risk of colorectal cancer development [16], however, the sample size of this study is so small(only 202 cases and 212 controls) that there is very limited impact on the overall result from the meta-analysis. In addition, IGFBP functions normally as an inhibitor of IGF’s action by blocking the binding of IGF to its receptor, but it can enhance the IGF’s action by protecting IGF from degradation under certain circumstance [25]. This may be another reason to explain the inconsistent findings among studies.

Previous studies revealed that *IGFBP3* A-202C polymorphism was associated with circulating IGFBP3 concentration, and the potential mechanism is that the C allele of the A-202C variant decreased promoter activity, affecting IGFBP3 transcription. A few meta-analysis studies also showed that this variant is associated with increased breast and prostate cancer risk [14], [15]. Our result is inconsistent with these two meta-analysis studies. We may not have detected an association between *IGFBP3* A-202C polymorphism and colorectal cancer for several reasons. First, the current knowledge of colorectal carcinogenesis indicates a multi-factorial and multi-step process that involves various genetic alterations and environmental factors. Some environmental factors, however, may predominate in the development of cancer, such as living habits and exposure to carcinogens. Without considering these factors, it may lead to the failure to detect the role of this polymorphism in cancer development. Second, the IGF axis includes the polypeptide ligands IGF1 and IGF2, the IGF receptors, and six binding IGF proteins (IGFBP1-IGFBP6), there are relation between many members, some single-nucleotide polymorphisms of familiar members, such as polymorphisms of, IGFBP2 and IGFBP3, may exert their complex and interacting functions with each other, which could affect the effects of A-202C polymorphism in the pathogenesis of cancer. Therefore, other polymorphisms as cancer risk factors should be taken into account to conclude a true effect. Third, the number of current case control studies is relatively small (only including five studies), we may have insufficient statistical power to generate an real risk estimation.

A few studies have confirmed that IGFBP-3 levels are influenced by the -A202C *IGFBP3* polymorphism [11], [26], and this polymorphism could influence responsiveness to growth inhibitors whose action involves up-regulation of IGFBP3 and the efficacy of various agents proposed for cancer chemoprevention [11]. A difficult issue for clinicians is determing which subpopulations are more sensitive to chemoprevention. If large sample studies could explore the association between *IGFBP3* polymorphisms and colorectal cancer, *IGFBP3* may provide an example of a gene whose polymorphic variation is relevant to the pharmacogenomics of cancer prevention.

Assessment of heterogeneity is necessary for most meta-analyses. Heterogeneity could result from genotyping error, population stratification, selection bias, gene-environment interaction, or chance. There is no significant heterogeneity in IGFBP3 A-202C and Gly32Ala genotype comparisons (see Table 2), and meta-analysis results showed that there were no significant effect between IGFBP3 A-202C, Gly32Ala polymorphisms and colorectal cancer. We concluded that subgroup analysis is not necessary in the present study.

Although we have put considerable effort and resources into testing the possible association between *IGFBP3* polymorphisms and colorectal cancer risk, there are still some limitations inherited from the published studies. First, some non-differential misclassification bias is possible. One hospital based case control study selected hospital patients without colorectal cancer as the reference group. Therefore, non-differential misclassification bias is possible because the study may have included the control group who has different risk of developing colorectal cancer. Second, we can not perform subgroup analysis for specific cancer sites because of limited information from original studies; for example, patients in only one article were divided by colon and rectum [22].

In conclusion, this meta-analysis suggests that *IGFBP3* A-202C and Gly32Ala polymorphisms may not be associated with colorectal cancer development. However, it is necessary to conduct large sample studies using standardized unbiased genotyping methods and well matched controls. Such studies taking these factors into account may eventually lead to a better, comprehensive understanding of the association between the polymorphisms in the GH-IGF pathway and colorectal cancer risk.

## Supporting Information

Table S1 **PRISMA checklist.** This table described reported page number of each necessary section of Meta-analysis according to PRISMA Statement.
(DOC)
